# An Entry/Gateway^® ^cloning system for general expression of genes with molecular tags in *Drosophila melanogaster*

**DOI:** 10.1186/1471-2121-10-8

**Published:** 2009-01-29

**Authors:** Omar S Akbari, Daniel Oliver, Katie Eyer, Chi-Yun Pai

**Affiliations:** 1 Biology Department, University of Nevada, Reno, 1664 N. Virginia Street, M/S 314, Reno, NV 89557, USA

## Abstract

**Background:**

Tagged fusion proteins are priceless tools for monitoring the activities of biomolecules in living cells. However, over-expression of fusion proteins sometimes leads to the unwanted lethality or developmental defects. Therefore, vectors that can express tagged proteins at physiological levels are desirable tools for studying dosage-sensitive proteins. We developed a set of Entry/Gateway^® ^vectors for expressing fluorescent fusion proteins in *Drosophila melanogaster*. The vectors were used to generate fluorescent CP190 which is a component of the *gypsy *chromatin insulator. We used the fluorescent CP190 to study the dynamic movement of related chromatin insulators in living cells.

**Results:**

The Entry/Gateway^® ^system is a timesaving technique for quickly generating expression constructs of tagged fusion proteins. We described in this study an Entry/Gateway^® ^based system, which includes six P-element destination vectors (P-DEST) for expressing tagged proteins (eGFP, mRFP, or myc) in *Drosophila melanogaster *and a TA-based cloning vector for generating entry clones from unstable DNA sequences. We used the P-DEST vectors to express fluorecent *CP190 *at tolerable levels. Expression of *CP190 *using the UAS/Gal4 system, instead, led to either lethality or underdeveloped tissues. The expressed eGFP- or mRFP-tagged CP190 proteins are fully functional and rescued the lethality of the homozygous *CP190 *mutation. We visualized a wide range of CP190 distribution patterns in living cell nuclei, from thousands of tiny particles to less than ten giant ones, which likely reflects diverse organization of higher-order chromatin structures. We also visualized the fusion of multiple smaller insulator bodies into larger aggregates in living cells, which is likely reflective of the dynamic activities of reorganization of chromatin in living nuclei.

**Conclusion:**

We have developed an efficient cloning system for expressing dosage-sensitive proteins in *Drosophila melanogaster*. This system successfully expresses functional fluorescent CP190 fusion proteins. The fluorescent CP190 proteins exist in insulator bodies of various numbers and sizes among cells from multiple living tissues. Furthermore, live imaging of the movements of these fluorescent-tagged proteins suggests that the assembly and disassembly of insulator bodies are normal activities in living cells and may be directed for regulating transcription.

## Background

**Figure 1:**
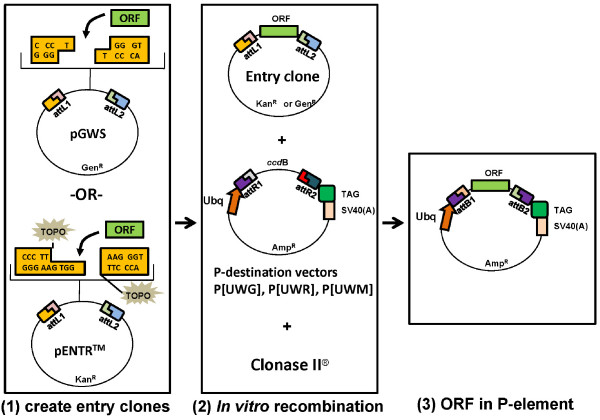
**The Entry/Gateway^® ^cloning procedures for generating epitope-tagged fusion proteins using the P-element destination vectors**. The procedures of Entry/Gateway^® ^cloning are illustrated in the diagram as two major steps. (1) In the first step, a fragment encoding the open reading frame (ORF) is inserted into an entry vector to generate entry clones. Two entry vectors were described in this study: (*i*) the pGWS which uses a TA-based method; (*ii*) pENTR/D-TOPO (Invitrogen) which uses a TOPO-based method. (2) In the second step, the ORF in the entry clone is recombined into one of the P-DEST vectors.

The "Entry/Gateway^®^" technology is a recently-developed plasmid construction strategy for rapidly cloning one DNA sequence into multiple destination plasmids. This technology greatly reduces the labor-intensive and time-consuming procedures of classical plasmid construction. It is particularly useful to create multiple plasmids for expressing various tagged versions of a specific protein or for expressing the protein under various promoters. To use this technology, first a donor plasmid containing the DNA of interest is created, known as an "entry" clone. Subsequently, the desired DNA in the entry clone is recombined, in an *in vitro *recombinase reaction, into a variety of destination vectors (figure 1). Due to the precision of the recombinase reaction, the desired sequence is inserted at the designated position of the destination vector. This makes the technology useful in many applications, for example generating epitope-tagged fusion proteins in which controlling the reading frame is critical.

Fusion proteins with molecular tags are widely used in biological studies. The most widely used are green fluorescent protein (GFP) and red fluorescent protein (RFP) for their visualization of tagged proteins in living cells. Other tags are also commonly used, such as the epitope tags myc, FLAG and HA. Each tag has its specific benefits and disadvantages, and it is desirable to generate multiple plasmids for producing various tagged versions of a protein. Occasionally, a tagged protein might function differently than the original protein. To resolve this potential problem, one may place the tag at either the N-terminal or C-terminal region of the protein or try other tags in a trial-and-error manner. A series of destination vectors containing various combinations of a promoter and molecular tags can greatly reduce the time and labor of creating the required plasmids in these applications. In *Drosophila*, the P-element based UAS/Gal4 system is widely used for expression of transgenes due to its extremely versatile nature [1]. An extensive set of UAS P-element destination vectors have been created previously, for example, the pPWG and pPWR vectors, which can express the eGFP- or mRFP-tagged fusion proteins (T. Murphy personal communication,  and ). The UAS/Gal4 system, however, may cause over-expression of the desired protein, which often induces unexpected effects when dosage-sensitive genes are studied, for example the chromatin insulator protein CP190 described in this study. We have developed, based on the Entry/Gateway^® ^strategy, a cloning system that uses the ubiquitin (Ubi-63E) promoter to express genes at lower and tolerable levels and can be used to quickly generate plasmids for expressing tagged fusion proteins in *Drosophila melanogaster*.

Chromatin insulators are a class of regulatory DNA elements in the genome. Protein complexes assembled on a chromatin insulator sequence can insulate enhancers from a promoter when the insulator is located between the enhancers and the promoter. Proposed functions of chromatin insulators include organizing boundaries of chromatin sub-domains and regulating local gene expression by interfering with enhancer-promoter interactions. It has been hypothesized that chromatin insulator complexes, bound at distal genetic locations, may "aggregate" to form structures named "insulator bodies"[2,3]. We have used the developed cloning system to express CP190 in *Drosophila melanogaster*. CP190 is a nuclear protein in the fly and is a shared essential component of several kinds of chromatin insulator complexes [3-5]. The fluorescence of the CP190-eGFP or CP190-mRFP proteins allows us to monitor the distribution of related chromatin insulators in living cells. We discovered that the distribution of CP190-related chromatin insulators varied significantly in living cells from separate tissues. We also observed significant movement of CP190-containing insulator bodies in living cells, which resulted in the fusion of smaller aggregates into larger complexes. This activity likely reflects the alterations of the organization of chromatin higher-order structure in these cells.

## Results

### A vector for generating entry clones using TA-based methods

**Figure 2:**
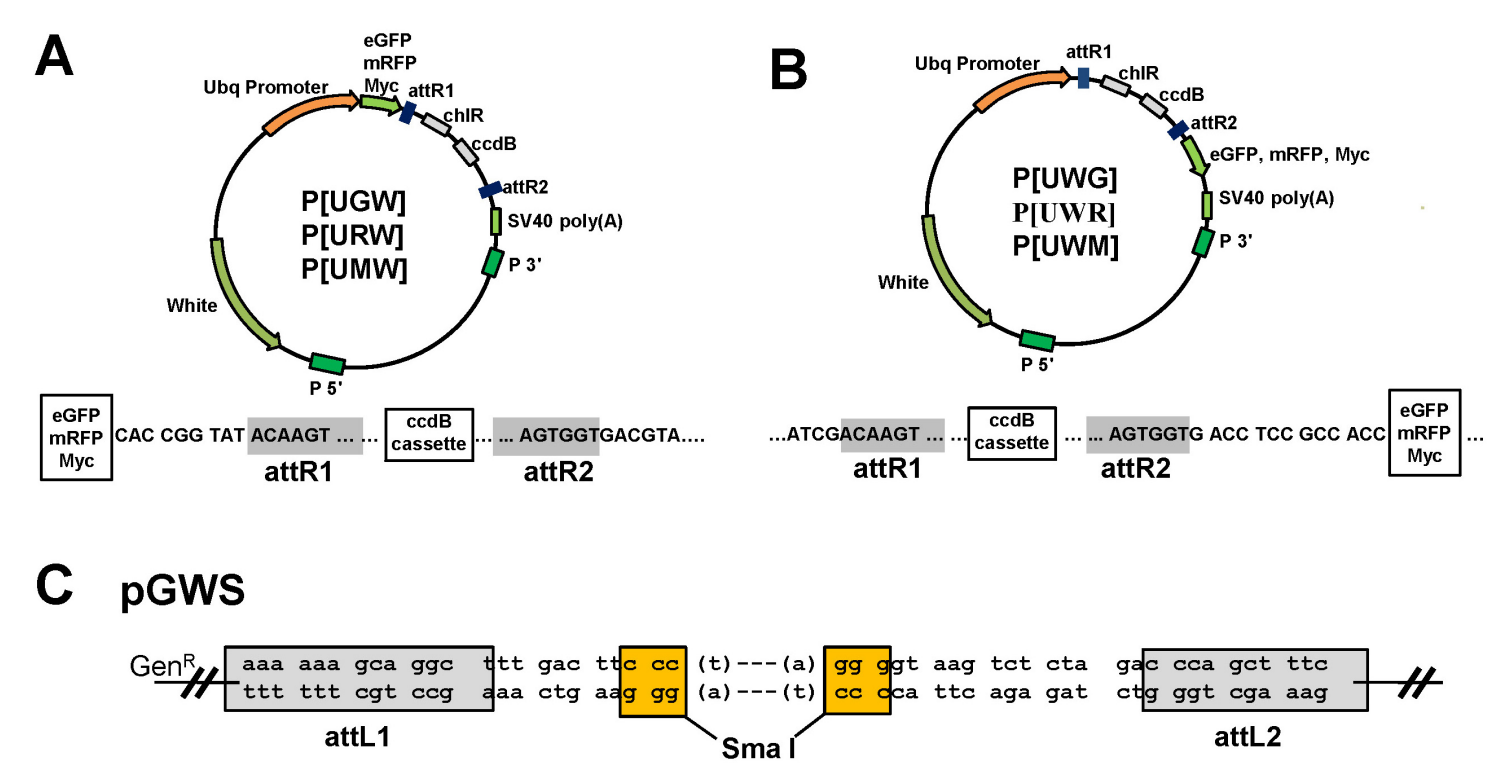
**Structure of the pGWS and P-destination vectors**. (A-B) The detailed structure of P-DEST vectors with N-terminal tags (A), and with C-terminal tags (B). The sequences and the reading frame from the epitope tags are shown below the map. The attR1 and attR2 sequences for Clonase II^® ^recombination are shaded. (C) The structure and sequences of the pGWS. The circular pGWS has a unique SmaI site which is a restriction enzyme that can cut pGWS into a linear DNA with two blunted ends. The 3'-protruding ends after Taq T-tailing for TA cloning are shown in brackets. The reading frame after LR recombination with P-DEST vectors is indicated.

We have created a vector for generating entry clones named pGWS that uses Gentamicin (Gen) as the selectable marker. The pGWS is particularly useful for the cloning of unstable DNA sequences using the *SURE^® ^*strain of *E. coli *(Stratagene) which is Kanamycin (Kan) resistant. The pGWS is designed for a TA-based or blunt-end-based cloning method. Important features of pGWS include: (*i*) a restriction enzyme site (CCCGGG) that becomes blunt-ended after the SmaI digestion; (*ii*) attL1 and attL2 sequences flanking the SmaI site for LR recombination; (*iii*) Gen as the selectable marker; (*iv*) an insert in the first frame in pGWS will be in frame, after recombination, to the tags in all the P-DEST vectors described below (figure 2) and also in frame with the tags of the commercially-available destination vectors from Invitrogen. To use pGWS in TA-based cloning, pGWS is linearized by SmaI to become blunt-ended, followed by a Taq polymerase incubation to add a "T" overhang on the 3' ends.

**Figure 3:**
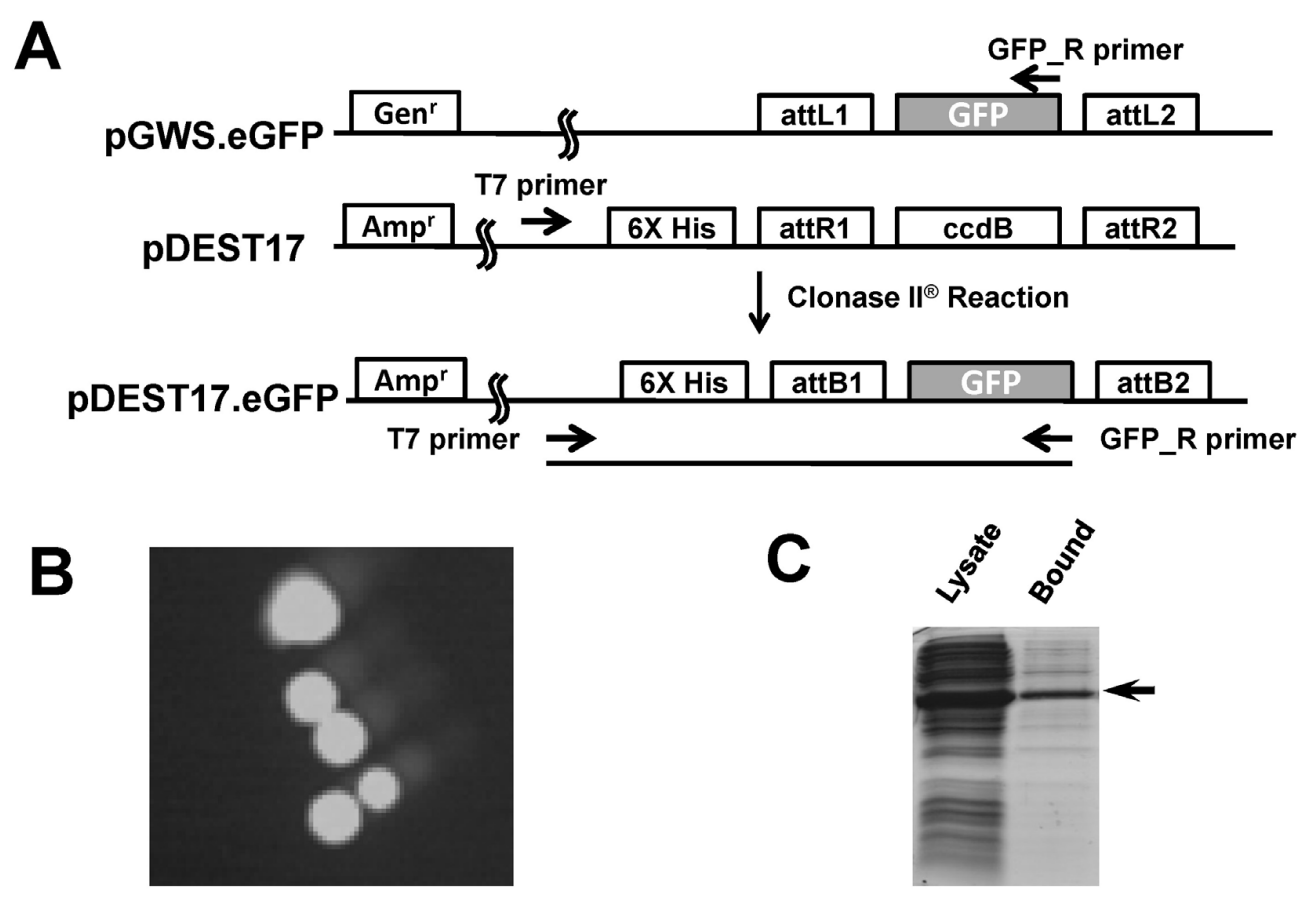
**The pGWS entry clones are efficient in the Clonase II LR reactions**. (A) The diagram of LR reaction of the pGWS.eGFP entry clone and pDEST17. Shown are features of the two parental plasmids and the recombined product encoding the eGFP protein with poly-His tag. The arrows indicate the primers for determining the bacteria colonies that contain the correctly recombined plasmid. (B) Colonies of the eGFP-His expressing bacteria are fluorescent. (C) The bacterial lysate and the His-bind-chromatography-purified eGFP-His protein were analyzed by SDS-PAGE and stained with Coomassie blue. The arrow points to the purified His-eGFP protein.

To test the efficiency of pGWS entry clones in the LR Clonase II™ recombination reactions, we inserted the eGFP sequence into pGWS and obtained the entry clone pGWS.eGFP, which was subsequently recombined with the pDEST17 Gateway^® ^vector (Invitrogen) in an LR Clonase II™ reaction (figure 3A). Two clones containing the eGFP insert were analyzed by sequencing and both clones encode the 6 × His-eGFP fusion protein in the predicted reading frame. The 6 × His-GFP fusion protein was fluorescent in bacteria (figure 3B) and can be purified by a His-binding Ni^2+ ^column (figure 3C). The result indicates that pGWS is efficient in the LR reaction. We compared the pGWS result with other LR reactions we performed with pENTR/D-TOPO (Invitrogen) entry clones, and the LR reaction efficiencies are very similar between pGWS-based and pENTR/D-TOPO-based entry clones (data not shown).

### A set of P-element based Gateway^® ^destination vectors for general expression of epitope-tagged fusion protein in flies

In addition to the entry vector, we also created a set of P-element destination vectors (P-DESTs) for expressing tagged fusion proteins in *Drosophila melanogaster*. Each P-DEST vector contains the *Ubi-63E *promoter, which can drive the expression of a transgene in many tissues. In addition, each P-DEST encodes one of the tags (eGFP, mRFP, or MYC), such that the fusion protein can be easily detected by self-fluorescence or by commercially-available antibodies to these tags. The features of these vectors include: (*i*) a CaSpeR backbone containing a *white*^+ ^(*w*^+^) selectable marker for selecting transformed flies and the Ampicillin selectable marker for propagation in *E. coli*; (*ii*) a *Ubi-63E *promoter that can drive the expression of a transgene in most tissues and is responsive to heat shock [6]; (*iii*) a Gateway^® ^cassette containing the *ccdB *gene as the selectable marker for negatively selecting the non-recombined P-DEST DNA after the *in vitro *recombinase reaction; (*iv*) an epitope tag, either eGFP, mRFP, or MYC that may be fused either to the N- or C-terminus of the transgene; (*v*) an SV40 poly(A) signal for stabilizing the mRNA in the cell to facilitate expression (figure 2A and 2B). The features of all of the P-destination vectors are summarized in table 1.

**Table 1:** The features of the P-DEST vectors and pGWS.

**Vector Name**	**Vector Type**	**Tag**	**Tag Location**	**Promoter**
pP(UWG)	P-element destination vector	GFP	N-Terminal	Ubiquitin
pP(UGW)	P-element destination vector	GFP	C-Terminal	Ubiquitin
pP(UWR)	P-element destination vector	RFP	N-Terminal	Ubiquitin
pP(URW)	P-element destination vector	RFP	C-Terminal	Ubiquitin
pP(UWM)	P-element destination vector	Myc	N-Terminal	Ubiquitin
pP(UMW)	P-element destination vector	Myc	C-Terminal	Ubiquitin
pGWS	TA Entry Vector	NA	NA	NA

### Over-expression of CP190 using the Gal4/UAS system causes lethality or developmental defects

**Figure 4:**
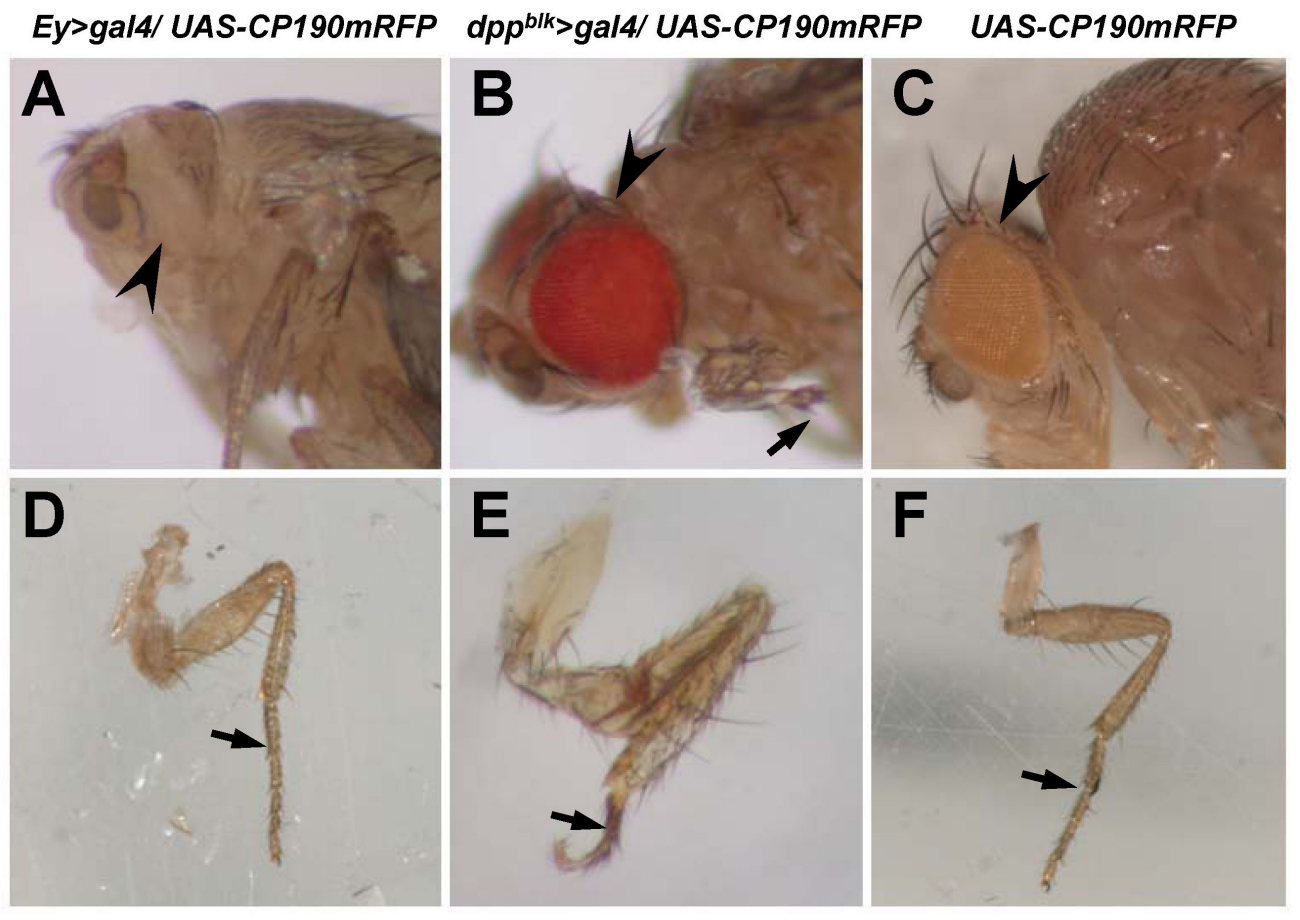
**Morphological phenotypes of CP190 over-expressingflies**. Morphological phenotypes of the *Ey *> *Gal4/UAS-CP190mRFP *fly (A and D), the *dpp*^*blk *^> *CP190mRFP *fly (B and E), and the *UAS-CP190mRFP *fly (C and F). (A and D) In *Ey *> *Gal4/UAS-CP190mRFP *flies, the eyes were not developed (A, arrowhead). The legs are normal (D, arrow). (B and E) In the *dpp*^*blk *^> *Gal4/UAS-CP190mRFP *flies, the eyes were developed, but were slightly rough (B, arrowhead). The distal parts of the legs, including tarsal and claw segments were underdeveloped or missing (B and E, arrows). (C and F) In the UAS-CP190mRFP flies, the eyes are normal (C, arrowhead). The legs are normal (F). All arrows point to the first tarsal segment of the first leg (B, D, E, and F).

The CP190 protein is expressed ubiquitously in almost all tissues in *Drosophila melanogaster*. It is present in at least two types of chromatin insulator complexes, including the Su(Hw) insulator complex[3] and the *CTCF *insulator complex[4]. Current evidence suggests that CP190 has important roles in the organization of chromatin and in the regulation of gene expression in the cell nucleus. To monitor the distribution of CP190-related chromatin insulator complexes in living cell nuclei during fly development, P-elements encoding eGFP- or mRFP-tagged CP190 (*UAS-CP190eGFP*, or *UAS-CP190mRFP *respectively) were created using the pPWG and pPWR vectors, which contain the UASp promoter. All flies carrying the UAS-CP190 transgenes are healthy but do not express the CP190 fusion protein (data not shown). To induce the fusion protein in flies, we crossed the *UAS-CP190eGFP *or *UAS-CP190mRFP *flies to the *act5c *> Gal4, which could drive the expression of the UAS transgenes in many tissues. Surprisingly, we found that the embryos containing both the *act5c *> Gal4 and the *UAS-CP190 *transgenes were lethal (data not shown). We crossed the UAS flies to the *ey *> *Gal4 *flies, which should drive the expression of fusion proteins in the developing eyes. The resulting flies did not develop eye tissues (figure 4A arrowhead), but other tissues, e.g. legs, are normal (figure 4D). We also crossed the *UAS-CP190 *flies to the *dpp*^*blk *^> *Gal4 *flies, which should drive the expression of fusion proteins in the developing leg tissues[7] and eye tissues[8]. The resulting flies did not have normal legs. The distal parts of the legs, including tarsal and claw segments, were underdeveloped or missing (figure 4B arrow, and 4E). The flies, however, developed close to normal eyes with a slightly rough shape (figure 4B arrowhead). These developmental defects are presumably due to over-expression of CP190 induced by *ey *> *Gal4 *or *dpp*^*blk *^> *Gal4*, since *UAS-CP190 *flies are morphologically normal (figure 4C and 4F). To see if mild induction of *CP190mRFP *expression may be achieved by *hs70 *> *Gal4*, we crossed *UAS-CP190mRFP *flies to *hs70 *> *Gal4 *flies. We could not detect any fluorescent signals in the resulting embryos and larvae. Most larval tissues in the 3^rd ^instar larvae were in their normal shapes, except that they did not have salivary glands of a detectable size (data not shown). This is likely due to the *hs70 *> *Gal4 *inducing the expression of the CP190 fusion proteins in developing salivary gland tissues, but not in other tissues, without heat shock. The expressed CP190, however, disrupted the development of salivary glands, although *CP190 *in the wildtype flies is normally expressed in the salivary gland cells [3,9]. To induce the general expression of CP190 fusion proteins, we treated the *hs70 *> *CP190mRFP *3^rd ^instar larvae with one dose of heat shock at 37°C for 20 mins. The treated larvae failed to pupate and the treatment resulted in lethality (data not shown). These unexpected results indicate that *Drosophila *is sensitive to the expression levels of CP190. Over-expression of *CP190 *in embryos and in larval imaginal discs severely disrupts development, although the CP190 protein in wildtype flies is expressed in all tissues from embryos to adults.

### Expression of CP190 using P-DEST vectors results in normal flies

**Figure 5:**
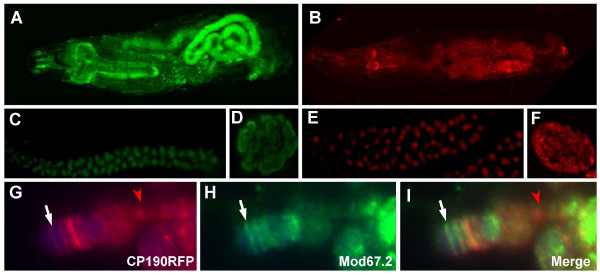
**Expression of GFP- or mRFP-tagged CP190 in transgenic flies**. (A-B) A third instar larva expressing eGFP-tagged CP190 (A), or mRFP-tagged CP190 (B). (C-D) Fluorescent signals of CP190eGFP in a salivary gland (C), and in the nucleus of a salivary gland cell (D). (E-F) Fluorescent signals of CP190mRFP in a salivary gland (E), and in the nucleus of a salivary gland cell (F). (G-I) The distribution of CP190mRFP (G) and Mod67.2 (H) proteins at the tip of X chromosome. The polytene chromosome was prepared from a *y*^2 ^3^rd ^instar larva. The white arrows point to the *y *locus where contains a copy of the *gypsy *insulator. The red arrow point to the location that contain only CP190 protein but do not contain Mod67.2 protein.

To circumvent the over-expression problems we experienced with the Gal4/UAS system, we used our P-DEST vectors (pUWG and pUWR). As described above, these vectors contain the *Ubi-63E *promoter for driving the eGFP- or mRFP-tagged fusion proteins. We obtained multiple transgenic lines from each P-element, including two mRFP lines and eight eGFP lines. In all transgenic lines, we detected eGFP or mRFP-tagged CP190 proteins. The fluorescent signals of CP190eGFP or CP190mRFP were observed in many tissues in all developmental stages, including embryos, larvae (figure 5), and adults. We next determined if the tagged CP190 proteins behave similarly to the wildtype CP190, which has been shown to be present on polytene chromosomes as many bands [3,9]. Both eGFP- and mRFP-tagged CP190 were detected, via their fluorescent signals, as many bands on polytene chromosomes in the nuclei of living salivary gland cells (figure 5C–5F). The bands of tagged CP190 were also detected on squashed polytene chromosome samples (figure 5G–5I). The CP190mRFP fusion protein co-localizes with Mod(mdg4)67.2, another protein in the *gypsy *complex, at the *gypsy *insertion site at the *yellow *(*y*) locus on the *y*^2 ^chromosome (figure 5G and 5I white arrows). This suggests that the tagged CP190 protein is recruited to the *gypsy *insulator complex. In addition to the bands containing *gypsy *insulator proteins, CP190mRFP also localizes to other Mod67.2-independent sites (figure 5G and 5I red arrows), the same as wildtype CP190 as reported previously [3]. The banding pattern of CP190mRFP proteins on polytene chromosomes was indistinguishable from that of the wildtype CP190 protein. The tagged-CP190 transgenes also rescued the lethality of the homozygous *CP190 *mutation (data not shown) and rescued the defective *gypsy*-dependent *y*^2 ^and *ct*^6 ^phenotypes of the homozygous CP190 mutation (explained in detail in a separate manuscript in preparation). All evidence indicates that the tagged CP190 proteins function similarly, if not exactly the same, as the wildtype CP190. Since the *Ubi-63 *promoter activity may be stronger with heat shock, we treated the larvae carrying the CP190mRFP transgene with one dose of heat treatment at 37°C for 20 mins and monitored the CP190mRFP signal from 2 hours post-treatment until 24 hours post-treatment. We detected only slightly elevated expression of CP190mRFP after 3 hours and no significant changes afterward, judging by the slightly increased fluorescent signal. The heat-treated larvae were viable and developed into normal flies (data not shown). This result indicates that the lethality of *hs-Gal4 *> *UAS-CP190mRFP *larvae after heat treatment described in the above section was not due to the activity of CP190mRFP during or after heat treatment but was likely due to over-expression induced by the *Gal4/UAS *expression system.

### Formation of "insulator bodies" in CP190mRFP-expressing cells

**Figure 6:**
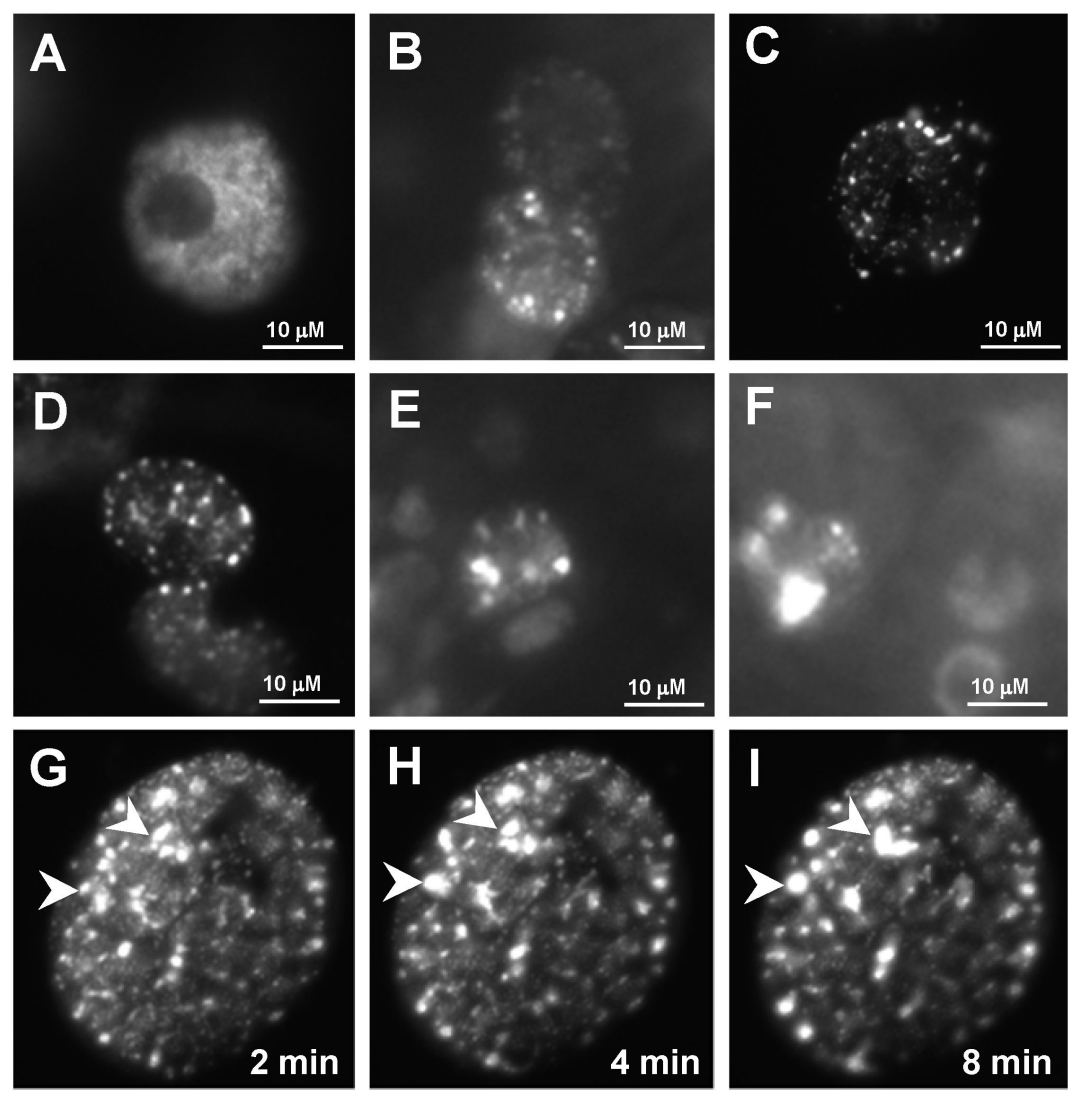
**Dynamic distribution of CP190-containing chromatin insulators in living cells**. (A-F) Fluorescent signals of CP190mRFP in living imaginal disc cells. (G-I) Movement of chromatin insulator bodies shown by CP190mRFP time-lapse images taken at the indicated times. Arrows point to insulator bodies which are moving toward each other (G and H) and the fused large insulator bodies (I).

We reported previously that insulator complexes containing CP190 in diploid cell nuclei exist as particles of various sizes. We proposed that this phenomenon was due to insulator complexes at separate genetic locations coming together and forming higher order structures, named "insulator bodies" [2,3]. To understand the organization of insulator bodies in living cells, we examined the distribution of CP190mRFP in cells of living larval tissues, including imaginal disc cells and brain cells. We found a wide range of distributions of CP190-containing insulator bodies among varying cell types (figure 6A–6F): some cells had thousands of tiny particles spreading all over the nuclei (figure 6A); some cells had around 10–30 bigger insulator bodies accompanied by many tiny ones (figure 6B–6D); and others had less than 10 big aggregates in the nuclei with a few tiny ones (figure 6E and 6F). We also noticed that many of the insulator bodies were localized at the periphery of the nuclei (figure 6B–6F), supporting the idea that these insulator complexes may be interacting with nuclear periphery substrates such as nuclear lamina. Both eGFP- and mRFP-tagged CP190 showed similar results. The results indicate that the formation of insulator bodies is a normal activity in living cells. The various distribution patterns are likely the result of unique organization of insulator bodies in the nuclei of each individual cell type. To further investigate the movement of insulator bodies in living cell nuclei, we monitored the fluorescent signals of CP190mRFP in living cells at several time points (figure 6G–6I). We found that insulator bodies were moving in the nucleus. We also observed that some smaller insulator bodies appeared to fuse to form larger insulator bodies (figure 6G-6I arrows). It is not yet known what mediates the movement of the insulator bodies; neither do we know the mechanism through which the assembly or disassembly of insulator bodies is regulated.

## Discussion

The genome projects of *Drosophila melanogaster *and of other species have revealed many genes that were not investigated before. Antibodies for the encoded proteins of these genes, in many cases, are not yet available. In such cases, it can be difficult to determine the biological activities of these proteins inside cells. Convenient methods for rapidly generating epitope-tagged transgenic *Drosophila melanogaster *lines will facilitate comprehensive *in vivo *functional analysis of these uncharacterized genes. *CP190 *is a housekeeping nuclear protein with general functions in chromatin organization. It is expressed in cells of all tissues in *Drosophila melanogaster*. Expression of *CP190 *in flies using the currently available *Gal4/UAS *induction system resulted in lethality or missing tissues. These unexpected results indicate that the development of *Drosophila melanogaster *is sensitive to the expression levels of *CP190*. We hypothesized that the available *Gal4 *drivers might have induced intolerably high expression levels of *CP190 *in cells from the *UAS-CP190 *transgene, thus leading to the developmental defects. We successfully corrected this over-expression problem by using the *Ubi-63E *promoter encoded in the P-DEST vectors. The *Ubi-63E *promoter has been characterized well previously [6,10-12]. The promoter can drive ubiquitous expression of a transgene in cells of most tissues. In addition, its temperature-sensitive feature allows for adjustment of the expression levels by heat treatment[13]. Flies expressing *CP190 *driven by *Ubi-63E *promoter are healthy and the expression rescued the lethality of the *CP190 *homozygous mutant. These results suggest that the promoters of *CP190 *and *Ubiquitin *may have similar strengths and may be interchangeable. Our results indicate that the P-DEST vectors allow the expression of CP190, a dosage-sensitive housekeeping gene, at tolerable levels in many tissues. These vectors will be valuable for studies of other proteins with similar dosage sensitivity. The six P-DEST vectors described in this paper provide time-saving tools for ubiquitous expression of fusion proteins, with an N-terminal or C-terminal epitope-tag, in *Drosophila melanogaster*. Recently, new technologies in transgenic vector design have been developed, such as the "MultiSite Gateway system" [14] and the *attB *site of the *phiC31 *system[15]. Incorporating these technologies into the P-DEST vectors in the future will make this vector system more flexible and easier to use.

Cloning of unstable DNA sequences, such as DNA with multiple repeated sequences or inverted repeats, is often performed in the *SURE^® ^*strain of *E. coli *(Stratagene) which is Kan resistant. Currently, a few vectors are commercially available for generating entry clones. They all use Kan as the selectable marker, such as pENTR™ vectors (Invitrogen). These vectors, thus, cannot be used for cloning with the *SURE^® ^*strain of *E. coli*. The newly-designed pGWS uses Gen as the selectable marker, thus avoiding this problem. The pGWS is unique from its parental plasmid pGWG[16], which uses an AhdI digestion to generate the 3' "T" overhangs for TA cloning. The AhdI-digested pGWG often loses the 3' "T" overhangs due to undefined exonuclease activities during the AhdI digesting reaction, causing low cloning efficiencies (data not shown). The redesigned pGWS significantly improves the cloning efficiency compared to the original pGWG vector.

Using P-DEST vectors, we have generated transgenic flies expressing CP190 proteins tagged with eGFP or mRFP. The CP190mRFP or CP190eGFP proteins can rescue the defective insulator function in homozygous *CP190 *mutations, suggesting that the mRFP- and eGFP-tagged CP190 proteins are fully functional in flies. The fluorescence of these tagged CP190 proteins allows us to view dynamic changes in the distribution of chromatin insulators in living cells. We detected, similarly to the antibody staining results published previously [3], the formation of insulator bodies in living cells. The number and sizes of insulator bodies, however, vary among cell types. Generally, insulator bodies are larger in cells that have fewer insulator bodies. This phenomenon suggests that large insulator bodies may be assembled from smaller ones. By monitoring the movement of CP190mRFP in living cells, we observed events of fusion, or assembly, of CP190-containing particles. These events reflected the reorganization of insulator bodies, and likely chromatin reorganization too, in the nucleus during the time of examination. The assembly and disassembly of insulator bodies appear to be normal activities within the cell nucleus, and, in addition, may be regulated differently in many cell types. Since most of the CP190mRFP fluorescent signals in polytene cells are associated with polytene chromosomes as many bands, it is likely that the majority of the CP190mRFP proteins in the diploid cells are also associated with DNA-bound chromatin insulator complexes. If most CP190 proteins are in DNA-bound complexes, the formation of big insulator bodies would be creating a higher-order chromatin structure that allows for the association of multiple insulator complexes at distant genetic locations. On the other hand, it is possible that the big insulator bodies are insulator complexes dissociating from a number of sub-regions of chromatin due to unknown regulatory mechanisms. It will be interesting to determine how cells with one pattern of chromatin insulator body distribution may be induced to change into another pattern and whether the rearrangement reflects, or causes, changes in local transcriptional activities. The assembly and disassembly of insulator bodies may be regulated via, for example, modifying the proteins in the insulator complexes to establish alternative higher-order structures of chromatin in separate cell types.

## Conclusion

We have developed a convenient cloning system using the Entry/Gateway^® ^technology. The cloning system includes one vector for generating entry clones and six P-element destination vectors (P-DESTs) for expressing fusion proteins in *Drosophila melanogaster*. The pGWS vector provides a non-commercial alternative method for creating entry clones. The vector will be particularly useful for cloning unstable DNA sequences using the *SURE^® ^*strain of *E. coli*. The six P-DEST vectors contain the *Ubi-63E *promoter, which can drive the expression of transgenes in many tissues in transgenic flies at physiological, or at least tolerable, levels. Each P-DEST also encodes one of the molecular tags that may be fused to either the N- or C-terminus of the transgenic protein. The pGWS and six P-DEST vectors provide time-saving tools for ubiquitous expression of fusion proteins in *Drosophila melanogaster*.

We have used the P-DEST vector system to express in flies the mRFP- or eGFP-tagged CP190, which is a shared essential component of multiple kinds of chromatin insulator complexes and is one of the dosage-sensitive housekeeping genes. The expressed CP190 fusion proteins function similarly to the wildtype CP190 protein. The fusion proteins associate with polytene chromosomes as multiple bands in living polytene cell nuclei. On squashed polytene chromosome samples, the tagged CP190 protein co-localizes with other proteins of the *gypsy *insulator complex at *gypsy *inserted loci. In living diploid cell nuclei, the fusion protein localizes to particles of various sizes, termed previously as "insulator bodies". By monitoring the fluorescent signals of CP190mRFP in living cells, we have found that CP190-containing insulator complexes are moving in the nucleus. In addition, we observed events of fusion, which presumably correlates to assembly, of CP190-containing insulator bodies of various sizes. This movement of insulator complexes may be a result of the altered organization of chromatin higher order structure. Our results indicate that the assembly and disassembly of insulator bodies are normal and dynamic activities in living cells.

## Methods

### Fly stocks

All fly stocks were maintained in 23°C or 26°C environmental insect culture chambers. The P-elements encoding tagged-CP190 were introduced into flies by the traditional germ-line transformation method[17]. The pPWG and pPWR vectors were obtained from *Drosophila *Genomic Resource Center. The *act5c *> *Gal4*, *ey *> Gal4, *dpp*^*blk *^> Gal4, and *hs70 *> Gal4 flies were obtained from Bloomington stock center.

### Tissue preparation, staining, microscopy and image processing

The eGFP- or mRFP-CP190 expressing larvae were viewed under a Leica MZ16 stereoscope and imaged using a Leica FX300 digital camera. For live insulator body imaging, eGFP- or mRFP-CP190-expressing tissues were dissected in phosphate saline. The dissected tissues were viewed immediately after dissection under a Leica DM5500 microscope and were imaged using a Leica FX350 digital camera. The spread polytene chromosomes were prepared and stained with indicated antibodies using a method described previously [3]. Rabbit-anti-GFP antibody (Invitrogen A11122) was used at 1:500 dilution. Mouse-anti-RFP antibody (Abcam) was used at 1:400 dilution. Adult flies were viewed under Leica S8 stereoscope. Images were taken by Leica DFC280 digital camera. Multiple pictures of one individual fly or of a tissue may be taken and overlaid for obtaining better depth of field. The image-overlay was processed automatically by Helicon Focus (Helicon Soft Ltd).

### Plasmid Constructions

The P-destination vectors were created using CaSPeR4 as the backbone. The KpnI-PstI fragment containing the *Ubi-63E *promoter in pWUM6 was inserted into the Kpn1/Pst1 sites of CaSPeR4 to become pP [CaSU]. The SV40(A) fragment was PCR amplified from vector pAWG (T. Murphy, unpublished results, obtained from the *Drosophila *Genomics Research Center) using the primer pair (forward primer 5'-GCGGCCGCCTAGCAGGATCTTTGTGAAG-3', reverse primer 5'-GCGGCCGCTGTTGAATACTCATACTCTTCC-3'). The resulting 976 bp fragment was digested with NotI and was inserted into the NotI site of pP [CaSU] to make p [CaSU(A)]. The gateway cassettes were digested from vectors pAGW, pAWG, pAMW, pAWM, pARW, pAWR (T. Murphy, unpublished results; obtained from the *Drosophila *Genomics Research Center) using restriction sites EcoRV/NheI and cloned into StuI/XbaI sites of p [CaSU(A)] to become pUGW, pUWG, pUMW, pUWM, pURW, pUWR respectively. For creating pGWS, pGWG was PCR amplified with the primer pair GWS_F (5'-GGGGTAAGTCTCTAGACCCAGCTTTC-3') and GWS_R (5'-GGGAAGTCAAAGCCTGCTTTT-3'). The resulting fragment was self-ligated with T4 ligase (NEB) and was propagated in the *SURE *strain of *E. coli*. To generate a TA-cloning vector from pGWS, pGWS DNA was digested with SmaI. The linearized DNA was recovered and a "T" was added at the 3' end of each strand by incubating the DNA with 2 mM of dTTP and Taq polymerase at 72°C for 2 hours. For generating the eGFP entry clone from pGWS, the eGFP fragment was PCR amplified from pUWG using the primer pair GFP_F (5'-CTTGTACAGCTCGTCCATGC-3') and GFP_R2 (5'-CTTGTACAGCTCGTCCATGC-3'). The resulting fragment was mixed with the pGWS TA vector DNA, prepared as described above, and was ligated with T4 ligase at 12°C overnight. The ligated product (pGWS.GFP) was propagated in the *Mach1 *strain (Invitrogen) of *E. coli*. For generating the His-tagged GFP fusion protein, the Clonase II^® ^reaction of pGWS.GFP and pDEST17 (Invitrogen) was performed following the instructions from Invitrogen.

## Abbreviations

eGFP: enhanced green fluorescent protein; mRFP: monomeric red fluorescent protein; Kan: Kanamycin; Gen: Gentamicin; P-DEST: P-destination vectors.

## Authors' contributions

OA constructed all P-DEST vectors, evaluated the distribution of CP190mRFP in transgenic flies, and determined the distribution and movement of CP190mRFP-containing insulator complexes in living cells and tissues. DO generated CP190eGFP transgenic flies, and determined the distribution of CP190eGFP and CP190mRFP on polytene chromosomes. KE constructed pGWS, generated pDEST17.eGFP expression plasmids using pGWS, and purified the eGFP protein from bacteria. CP generated the UAS-CP190mRFP and UAS-CP190eGFP flies, evaluated the phenotypes of all Gal4/UAS CP190 flies, and designed the P-DEST vectors and the pGWS.
